# Tumor cell apoptosis mediated by cytoplasmic ING1 is associated with improved survival in oral squamous cell carcinoma patients

**DOI:** 10.18632/oncotarget.1907

**Published:** 2014-04-17

**Authors:** Pinaki Bose, Satbir S. Thakur, Nigel T. Brockton, Alexander C. Klimowicz, Elizabeth Kornaga, Steven C. Nakoneshny, Karl T. Riabowol, Joseph C. Dort

**Affiliations:** ^1^ Department of Oncology, University of Calgary, HRIC 2A02, 3280 Hospital Drive NW, Calgary, Alberta, Canada; ^2^ Department of Biochemistry & Molecular Biology, University of Calgary, 3330 Hospital Drive NW, Calgary, Alberta, Canada; ^3^ Department of Population Health Research, Alberta Health Services – Cancer Care, 1403 - 29 Street NW, Calgary, Alberta, Canada; ^4^ Functional Tissue Imaging Unit, Translational Laboratories, Tom Baker Cancer Centre, 1331 - 29 Street NW, Calgary, Alberta, Canada; ^5^ Section of Otolaryngology-Head and Neck Surgery, Department of Surgery, University of Calgary, HRIC 2A02, 3280 Hospital Drive NW, Calgary, Alberta, Canada

**Keywords:** Apoptosis, AQUA, head and neck squamous cell carcinoma, ING1, oral squamous cell carcinoma

## Abstract

The ING1 epigenetic regulator and tumor suppressor plays a central role in apoptosis. The *Ing1* gene is functionally inactivated in many cancer types but is rarely mutated. Although most studies have implicated the major ING1 isoform, p33ING1b, in nuclear apoptotic signalling, we recently discovered a novel and potent apoptosis-inducing effect of p33ING1b translocation to the mitochondria in response to DNA damage. In the present study, we examined the impact of cytoplasmic/mitochondrial localization of p33ING1b in oral squamous cell carcinoma (OSCC) patient samples and explored the therapeutic potential of adenovirally-overexpressed p33ING1b in OSCC cell lines in combination with ionizing radiation (IR) treatment. In contrast with previous reports, we found that p33ING1b protein and mRNA levels are higher in OSCC compared to normal epithelial cells. In OSCC patient samples, higher levels of intra-tumoral cytoplasmic p33ING1b correlated with increased apoptotic markers and significantly better patient survival. This association was strongest in patients who received post-operative radiotherapy. IR treatment induced p33ING1b translocation to the mitochondria and adenoviral-p33ING1b synergized with IR to kill OSCC cells. Our results identify a novel functional relationship between cytoplasmic p33ING1b and patient survival and highlight the potential for the use of p33ING1b as a therapeutic agent in combination with adjuvant radiotherapy in OSCC.

## INTRODUCTION

The Inhibitor of Growth (ING) family of tumor suppressors (ING1-5) are an evolutionarily conserved group of plant homeodomain (PHD)-containing proteins with diverse functions including chromatin remodelling, DNA damage signalling, cell cycle regulation, cellular senescence and apoptosis. Until recently, all known apoptotic functions of the first member of the ING family, ING1, were restricted to the nucleus [1]. p33ING1b is the major ING1 isoform expressed in normal and cancer cells [2] (any further mention of ING1 in the text refers to p33ING1b). The role of ING1 in genotoxic stress-induced apoptosis is suggested by the accumulation of ING1 in nucleoli and its interaction with the proliferating cell nuclear antigen (PCNA) after UV-induced DNA damage [3, 4]. ING1 is a stoichiometric component of nuclear Sin3-HDAC complexes and the ING1 PHD domain can bind to H3K4Me3 marks on chromatin [5-7]. The simultaneous interaction of ING1 with HDACs and H3K4Me3 results in local histone deacetylation and inhibition of transcription, leading to DNA damage signalling and apoptosis [2]. Overexpression of ING1 increases pro-apoptotic BAX and p21 protein levels, alters the mitochondrial membrane potential and induces cell cycle arrest and apoptosis [2, 8, 9].

The *Ing1* gene is rarely mutated in cancer [10, 11]. However, decreased ING1 expression [8, 12, 13] and cytoplasmic mislocalization [11, 14], possibly mediated by 14-3-3 proteins and the tyrosine kinase Src [15, 16], have been proposed as mechanisms for the inactivation of ING1 nuclear function in many cancer-types [17]. We recently reported a novel extra-nuclear function of ING1; in response to DNA damage-inducing stimuli, ING1 translocated to the cytoplasm/mitochondria and induced apoptosis [1]. We also demonstrated that ING1 targeted to mitochondria using a mitochondrial targeting sequence is a more potent inducer of apoptosis than wild-type, predominantly nuclear ING1 [1].

Head and neck squamous cell carcinoma (HNSCC) is the sixth most common cancer world-wide [18]. Oral squamous cell carcinoma (OSCC) is a common HNSCC with a particularly grim prognosis. Approximately 264,000 patients are diagnosed with OSCC globally each year, including 30,000 in North America [18, 19]. As in other cancers, somatic mutations of the *Ing1* gene are rare in HNSCC [20]. Decreased levels of ING1 mRNA have been reported in HNSCC and in particular, OSCC [21, 22]. Cytoplasmic localization and decreased protein levels of ING1 have been reported to be associated with poor prognosis in HNSCC including OSCC [23, 24]. However, cytoplasmic localization of ING1 induces apoptosis [1], which, theoretically, could confer improved prognosis in patients with high levels of ING1 in their tumor cell cytoplasm. In the current study, we determined ING1 mRNA and protein levels in OSCC compared to normal oral cavity squamous epithelium (OCSE). We also investigated the intracellular distribution of ING1 in three OSCC cell lines and OSCC patient samples compared to normal OCSE. We determined the clinical relevance of ING1 intracellular distribution in OSCC. Finally, we demonstrated a synergistic apoptotic response to IR treatment after adenoviral delivery of ING1 in OSCC cell lines. Our experiments support the feasibility of delivering adenoviral ING1 to induce synergistic tumor cell killing in combination with IR, as a potential therapeutic strategy in OSCC.

## RESULTS

### ING1 expression and intracellular distribution in OSCC

**Figure 1:**
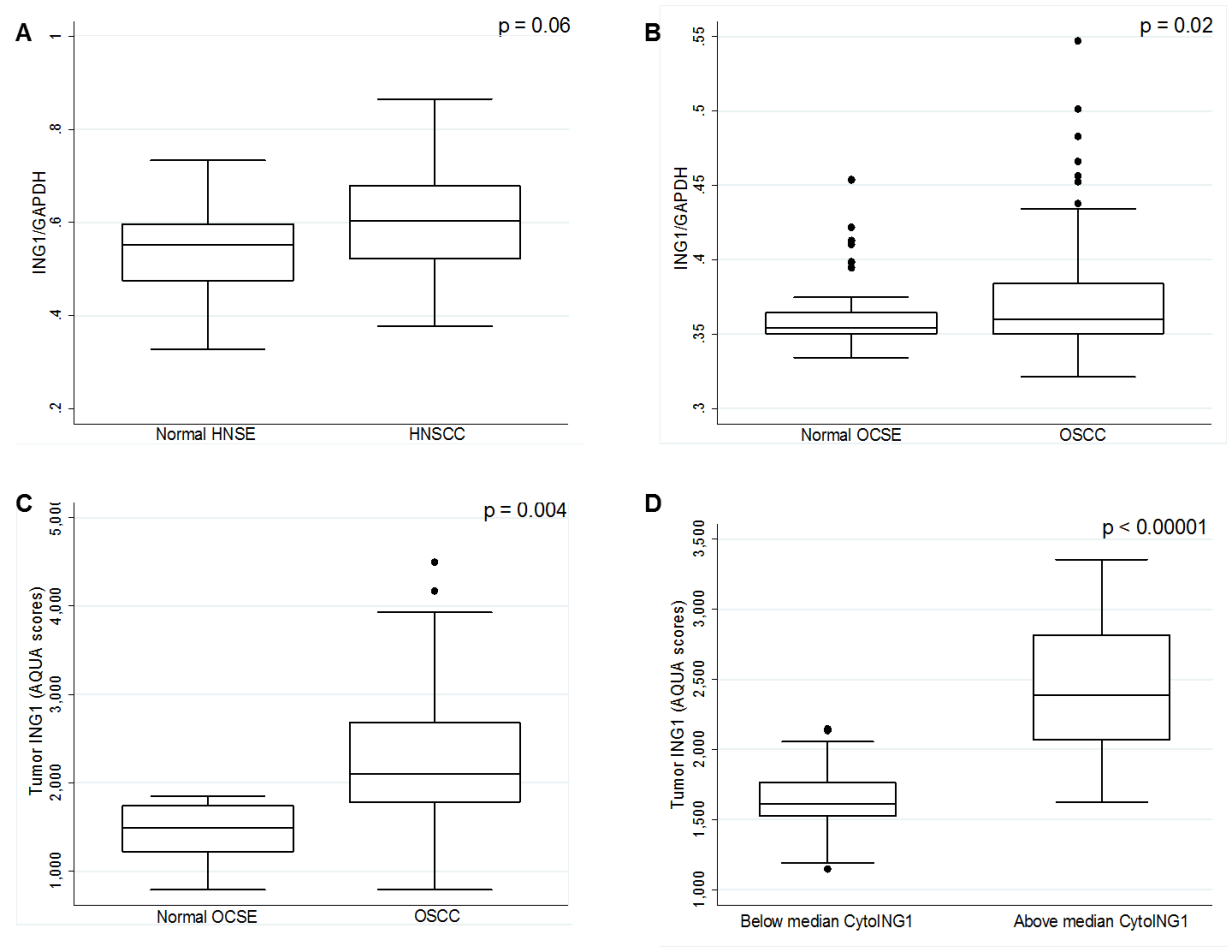
Analysis of ING1 mRNA and protein levels in HNSCC and OSCC (A) ING1 mRNA levels in 22 HNSCC patients with matched normal HNSE controls (GEO # GSE6631). (B) ING1 mRNA expression levels in 167 OSCC patients and in 45 matched OCSE controls (GEO # GSE30784). (C) ING1 protein levels in the 105 patient University of Calgary OSCC cohort compared to five matched normal controls. (D) ING1 protein levels compared in patients with below and above median cytoplasmic ING1 levels

*In silico* analysis of GEO datasets (#GSE30784 and #GSE6631) revealed that ING1 mRNA was expressed at a higher level in tumor samples compared to the corresponding normal tissue in two independent HNSCC and OSCC cohorts (Figure 1A and 1B). This observation, although contrary to previous reports [21, 22], was confirmed by fluorescence immunohistochemistry (IHC) and automated quantitative analysis (AQUA®) of OSCC tissue microarrays (Figure 1C). Additionally, tumors with relatively high total ING1 protein levels harboured a significantly higher amount of cytoplasmic ING1 (Figure 1D).

**Figure 2:**
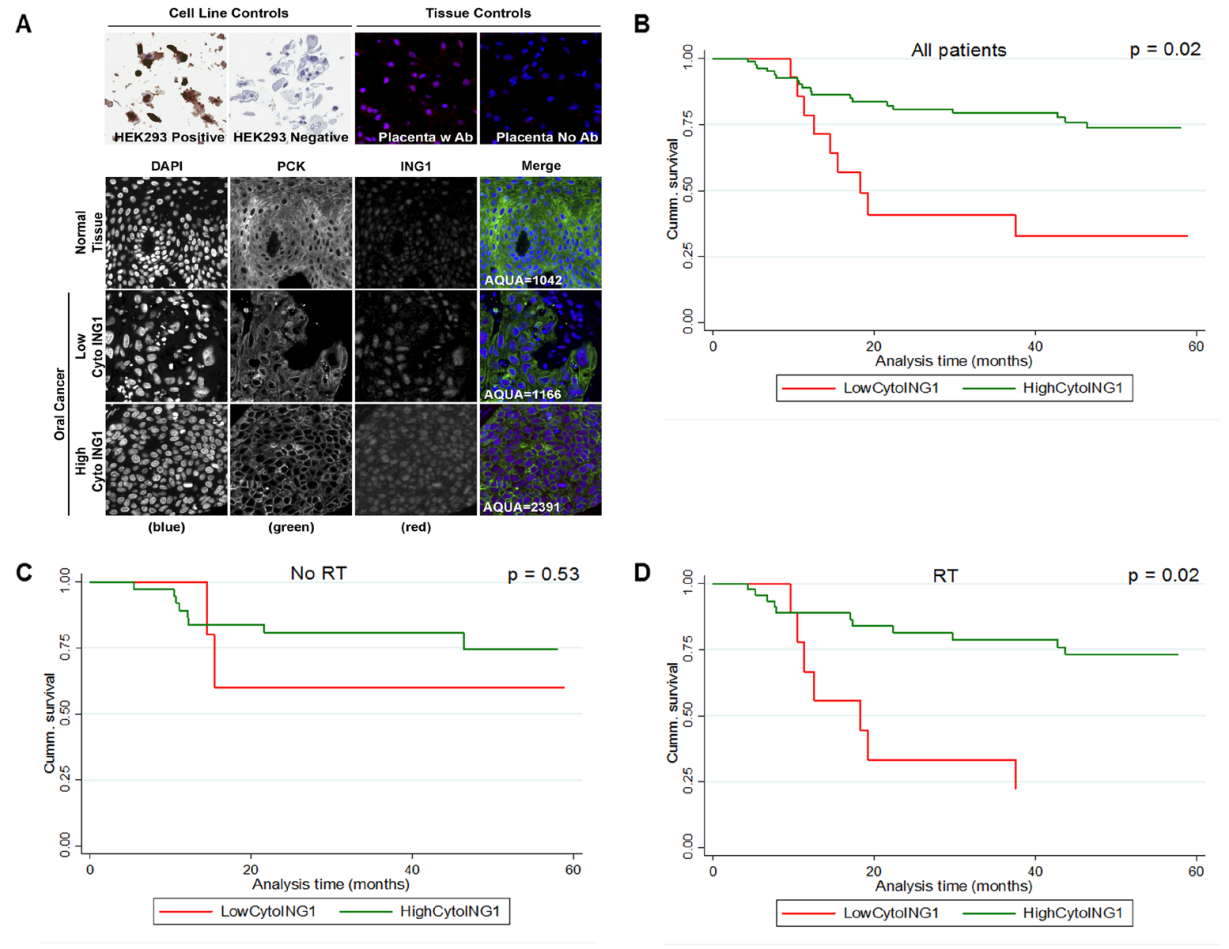
Association between survival and cytoplasmic ING1 levels in OSCC (A) Representative immnunofluorescence images of TMA cores stained for ING1 in normal OCSE and OSCC. Images were acquired at 20X magnification. Kaplan-Meier survival curves showing association between cytoplasmic ING1 and five year DSS (B) for the entire cohort, (C) for patients treated with surgery alone and (D) for patients treated with surgery followed by post-operative radiotherapy. Miller-Siegmund p-values are reported.

The intracellular distribution of ING1 in patient samples was of particular interest in light of our recent discovery that ING1 mediates apoptosis, in part, by translocating to the mitochondria [1]. We used AQUA® to objectively assess ING1 localization in our OSCC patient cohort. ING1 was predominantly nuclear in normal OCSE. Both nuclear and cytoplasmic ING1 were observed in OSCC (Figure 2A) and high cytoplasmic ING1 was associated with significantly improved disease-specific survival (DSS) (Figure 2B). The association between cytoplasmic ING1 and improved DSS was even stronger when the analysis was restricted to patients who received post-operative radiotherapy (Figure 2C and Figure 2D). In multivariate analysis, high cytoplasmic ING1 was an independent prognostic factor in our OSCC cohort after adjusting for pT-stage and pN-status (Table 2). Neither total tumor ING1 levels nor nuclear ING1 levels were associated with DSS (Figure S1).

### Cytoplasmic ING1 is associated with increased apoptosis

**Figure 3:**
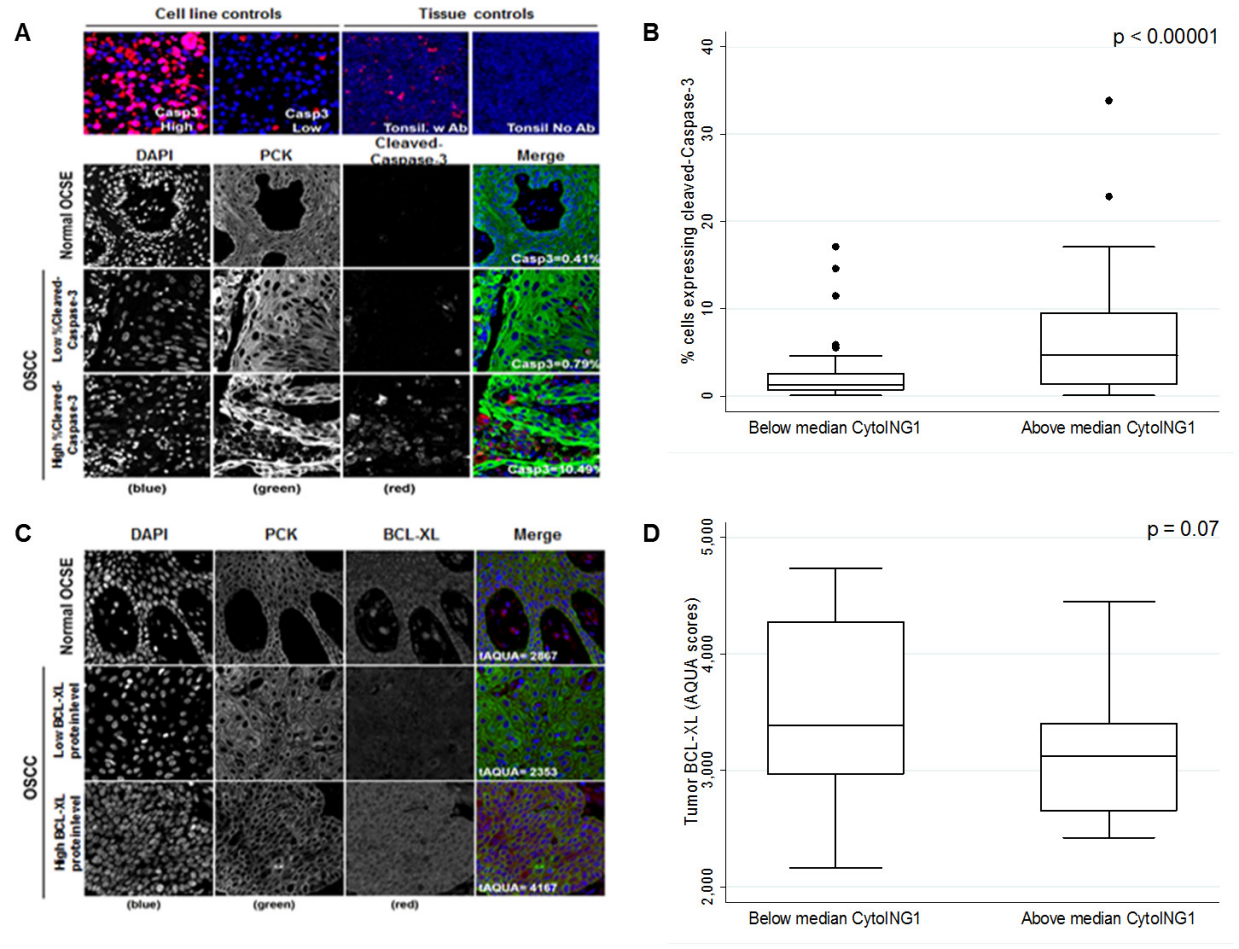
Association between cytoplasmic ING1 localization and apoptosis in OSCC patients (A) Representative immnunofluorescence images of TMA cores stained for cleaved-Caspase-3 in normal OCSE and OSCC. Images were acquired at 20X magnification. (B) Cleaved-Caspase-3 protein levels compared in patients with below and above median cytoplasmic ING1 levels. (C) Representative immnunofluorescence images of TMA cores stained for BCL-XL in normal OCSE and OSCC. Images were acquired at 20X magnification. (D) BCL-XL protein levels compared in patients with below and above median cytoplasmic ING1 levels.

The resolution afforded by AQUA® cannot definitively confirm mitochondrial localization. So, we used cytoplasmic localization as a proxy for mitochondrial localization and tested whether cytoplasmic ING1 correlated with cleaved-Caspase-3 levels in OSCC patient samples. Negligible cleaved-Caspase-3 levels were observed in normal OCSE by AQUA® (Figure 3A), but a range of cleaved-Caspase-3 levels were observed in OSCC (Figure 3A). OSCC samples with greater than median cytoplasmic ING1 exhibited high levels of cleaved-Caspase-3 (Figure 3B). However, there was no significant association between cleaved-Caspase-3 levels and DSS (p=0.70; Figure S2A). Staining of TMAs for the anti-apoptotic BCL-XL protein showed very low expression in normal tissue and a broad range of expression in OSCC samples (Figure 3C). High cytoplasmic ING1 was associated with decreased levels of BCL-XL protein (Figure 3D). We did not observe correlations between cytoplasmic ING1 and other BCL-2 family proteins such as BAX or BCL-2 (Figure S2B and S2C).

**TABLE 1:** Clinico-pathological characteristics of the University of Calgary OSCC cohort

	n = 105	LowCytoING1	HighCytoING1	Fisher's exact p-value
Gender				
Male Female	68 (64%) 37 (36%)	9 5	59 32	1.00
Age (years)				
Mean (SD)	59.98 (13.04)	55.55 (15.09)	60.46 (12.60)	0.60
Smoking History				
Never Ever Missing	27 (26%) 77 (73%) 1 (1%)	4 10 0	23 67 1	0.62
Alcohol History				
Never Ever Missing	11 (10%) 56 (53%) 38 (37%)	2 9 3	9 47 2	0.72
Pathological T-Stage				
pT1/pT2 (low) pT3/pT4 (high) Missing	59 (56%) 44 (42%) 2 (2%)	7 7 0	52 37 2	0.68
Nodal Status				
N0 Neck N+ Neck	61 (62%) 39 (38%)	7 7	57 34	0.36
Tumour Differentiation				
Well Moderate Poor Not stated	14 (13%) 55 (52%) 12 (12%) 24 (23%)	1 9 3 1	13 46 9 23	0.09
Treatment				
Surgery Surgery + RT Surgery + CRT	34 (32%) 67 (64%) 4 (4%)	5 7 2	29 60 2	0.08

**TABLE 2:** Univariate and Multivariate (Cox proportional hazards) analysis of five-year disease-specific survival

	Univariate		Multivariate	
	Hazard Ratio (95% C.I.)	p-value	Hazard Ratio (95% C.I.)	p-value
Cytoplasmic ING1 (high vs. low)	0.345 (0.158 - 0.750)	0.007*	0.338 (0.153 – 0.746)	0.005*
pN-status (pN0 vs. pN1/pN2)	2.173 (1.083 - 4.357)	0.029*	2.250 (1.104 – 4.588)	0.026*
pT-Stage (pT1/pT2 vs. pT3/pT4)	1.005 (0.986 -1.024)	0.560	1.011 (0.991 – 1.031)	0.263

* significant p-values

### Genotoxic stress-induced mitochondrial translocation of ING1

Since mitochondrial localization correlates with the ability of ING1 to induce apoptosis [1], we tested whether ING1 localizes to the mitochondria in OSCC cell lines in response to IR treatment. We observed robust mitochondrial translocation as determined by co-localization with MitoTracker® in CAL-27, UMSCC1 and UMSCC14B cells in response to IR (Figure S3). Peak mitochondrial localization of ING1 was observed in response to 2Gy of IR after 6hrs.

### IR synergises with ING1 to induce cell killing

**Figure 4:**
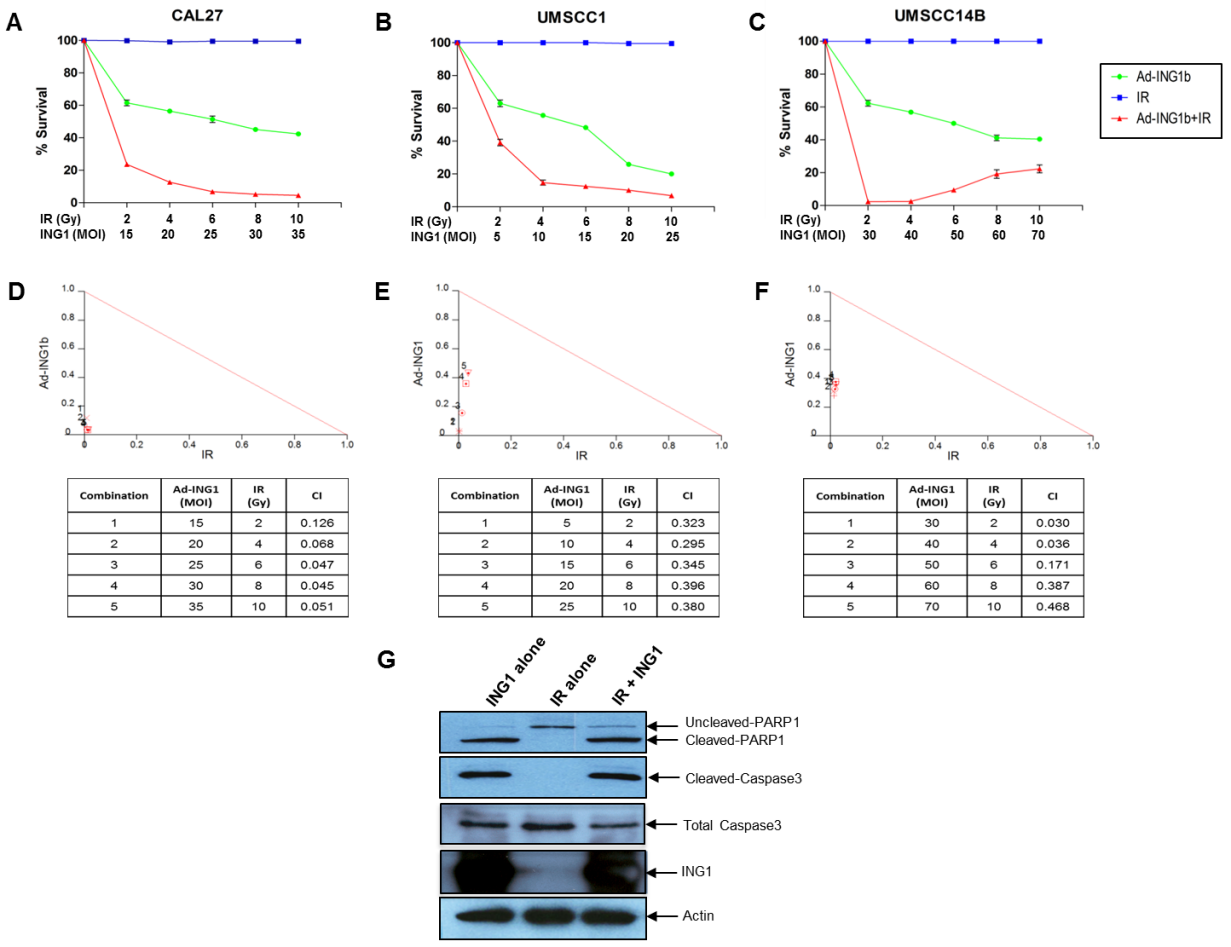
Cell death in OSCC cells in response to IR and ING1 (A) CAL-27 (B) UMSCC1 and (C) UMSCC14B cells were infected with indicated MOI of Ad-ING1 alone or in combination with indicated doses of IR. For CAL-27 and UMSCC1 cells, IR treatment was performed 12hrs post ING1 infection and cells were harvested 12hrs after IR treatment. For UMSCC14B cells, IR treatment was performed 24hrs post ING1 infection and cells were harvested 24hrs after IR treatment. Cell death was analyzed using the MTT assay. Isobolograms and CIs generated using the CalcuSyn software are shown for (D) CAL-27, (E) UMSCC1 and (F) UMSCC14B cells. (G) Western blot analysis of PARP1 and Caspase-3 cleavage in CAL-27 cells. Actin was used as a loading control.

Since both overexpression of ING1 and IR-induced mitochondrial translocation lead to apoptosis [1], we tested whether combining IR treatment and ING1 overexpression could synergistically impact cell killing. Infection using Adenoviral-ING1 (Ad-ING1) reduced the viability of CAL-27, UMSCC1 and UMSCC14B cell lines (Figures 4A-C). We found that IR alone did not induce cell death in any of the three cell lines (Figures 4A-C) within the time frame of these experiments (12 hours for CAL-27 UMSCC1 and 24 hours for UMSCC14B). However, as shown in Figures 4D-F, ING1 and IR did exhibit strong synergy at all MOIs and doses of IR. Caspase-3 and PARP1 cleavage were observed in cells infected with Ad-ING1 (Figure 4G), consistent with apoptosis causing decreased survival.

## DISCUSSION

In contrast to previous reports, we find that HNSCC and OSCC express higher levels of ING1 than normal squamous epithelium (Figure 1). This may be due to the fact that we compared levels in normal and cancer cells that were stained within the same microscopic fields of view. Investigation of ING1 levels in three independent cohorts using two completely independent platforms (Affymetrix gene array and AQUA®) gave consistent results in these cancers. Since ING1 is a stoichiometric member of HDAC complexes, this observation suggests that abnormal ING1 stoichiometry may promote neoplastic transformation. While downregulation of ING1 might promote oncogenesis in certain cancers, overexpression of ING1 might also lead to malignancy by disrupting the function of HDAC complexes.

Most studies examining ING1 localization have proposed that cytoplasmic localization compromises the tumor suppressive function of ING1 and represents a mislocalized pool of a normally nuclear protein [2, 25]. Our results challenge this dogma. Cytoplasmic ING1 was an independent prognostic marker in OSCC, after adjusting for pathological T-stage (pT-stage) and nodal status (pN-status). The strong correlation between the expression of apoptotic proteins (increased cleaved-Caspase-3 and decreased BCL-XL expression) and cytoplasmic localization of ING1 in tumors, combined with the significantly improved prognosis observed in patients with high cytoplasmic ING1, suggests a functional role for ING1 in mitochondria-mediated apoptosis. We also observed that an increase in total tumor ING1 protein levels is associated with a concomitant rise in cytoplasmic ING1 levels in OSCC samples (Figure 1D). Based on the observation that high cytoplasmic ING1 confers improved prognosis, high ING1 levels in OSCC tumors should confer a favourable prognosis because of increased mitochondrial ING1 localization and the promotion of apoptosis. However, in this study, total tumor (or nuclear) ING1 was not associated with prognosis (Figure S1A and B), which might indicate that ING1 abundance is not the only mechanism driving mitochondrial localization. Indeed, we found that cytoplasmic ING1 was also present in some OSCC tumors with low ING1 protein levels (Figure 1D).

This is the first study to measure ING1 protein levels in human tumor tissue using fluorescence IHC and AQUA® technology. AQUA® provides highly reproducible estimates of protein levels by minimizing observer bias and by incorporating several quality control measures. AQUA® also enables the measurement of protein levels in distinct subcellular and tissue compartments (e.g. nucleus/cytoplasm and tumor/stroma), providing additional insights into protein function and possibly improving the clinical applicability of this technique [26]. The inclusion of adjacent normal tissue in our TMAs serves as a control for estimating ING1 expression in normal OCSE. Cell lines expressing endogenous and ectopically high levels of ING1 were also included in TMAs for optimal antibody characterization.

The prognostic impact of cytoplasmic ING1 was strongest in patients who received post-operative radiation. Based on our observation that both ING1 overexpression and IR-induced ING1 translocation to the mitochondria lead to apoptosis (Figure S3) [1], we hypothesised that ING1 overexpression can be used to augment radiation sensitivity in OSCC. Indeed, ING1 synergized with IR to kill OSCC cells, despite all three cell lines being resistant to IR in the absence of ING1. IR resistance may be attributed to the short duration of incubation after IR treatment necessary for performing the combination studies (12hrs for CAL-27 and UMSCC-1, 24hrs for UMSCC-14B). Also, the evident lack of synergy between ING1 and IR in western blot experiments (Figure 4G; similar cleaved-caspase 3 and cleaved-PARP1 levels in “ING1 alone” and “ING1 + IR samples”) may be due to saturation in the level of detection of caspase-3 and PARP1 cleavage by western blot analysis. Alternatively, ING1 and IR may contribute to inducing cell death by pathways that are not dependent upon caspase cleavage in addition to activating pathways that are. The synergistic cell killing borne out by the isobolograms might reflect the pooled contributions of ING1 overexpression disrupting HDAC complexes in the nucleus and ING1 localizing to the mitochondria in response to genotoxic stress [1]. Likewise, the greater effectiveness of RT in OSCC patients with high cytoplasmic ING1 might reflect the more potent induction of apoptosis in these patient's tumors (Figure 2D).

Overall, these data show obvious parallels with the transcription-independent, mitochondrial functions of the p53 tumor suppressor [27]. Since we previously reported that mitochondrial translocation of ING1 is independent of p53 [1], the mechanism responsible for translocation needs further investigation, but it may involve binding of ING1 by the 14-3-3 family of proteins, promoting their export from the nucleus [15].

Our results show that in addition to effecting apoptosis through the nuclear machinery, ING1 can manifest an apoptotic function outside the nucleus similar to the pathway utilized by p53 to promote apoptosis in a transcription-independent manner [27]. Our data also suggest that adenoviral delivery of ING1 could act as an effective radiosensitizer in OSCC therapy.

## MATERIALS AND METHODS

### *In silico* analysis

The Gene Expression Omnibus (GEO) database was queried to compare gene expression data in 167 OS*C*C tumors with 45 normal tissue samples (GEO # GSE30784) and 22 HNSCC tumors with matched normal tissue (GEO # GSE6631). ING1 mRNA expression results were extracted from normal and cancer tissue in both datasets. Glyceraldehyde 3-phosphate dehydrogenase (GAPDH) expression was used as an internal control. Welch's t-test was used to assess significance.

### Patient cohort

Surgically resected samples from 121 histologically-confirmed OSCC patients were used in this study. Eligibility criteria included no prior history of HNSCC and treatment with primary surgery; adjuvant radiotherapy was used based on surgical pathology. The Tri-council Policy Statement for Research with Human Subjects (Canada) guided the conduct of the study and ethics approval was obtained from the University of Calgary Conjoint Health Research Ethics Board. Adequate FFPE tissue for Tissue microarray (TMA) construction was available for 105 of the 121 OSCC patients. Clinico-pathological characteristics of the patient cohort are provided in Table 1.

### Fluorescence IHC of TMAs

TMA construction has been described previously [28]. Image inquisition and analysis explained in next section. Protein levels were quantified for ING1, cleaved-caspase-3, BAX, BCL-2 and BCL-XL. Staining protocols for BAX, BCL-2 and BCL-XL have been described earlier [28]. For ING1 and cleaved-Caspase-3 staining, heat-induced epitope retrieval was performed by heating slides to 124°C in a citrate-based Target Retrieval Solution (pH 6.0) (Dako, Burlington, ON, Canada) for 6 minutes in a decloaking chamber (Biocare Medical, Concord, CA, USA). For ING1, TMA slides were incubated in a 1:200 dilution of the CAb2 mouse monoclonal anti-human antibody in SignalStain® Antibody Diluent (Cell Signaling Technology, Danvers, MA, USA), for 60 minutes at room temperature (RT). After 3 washes in 1x Tris-buffered saline and Tween 20 (TBST; Dako), slides were incubated with the mouse Envision+ HRP system (secondary antibody, Dako) for 60 minutes at RT. Slides were washed 3-times and incubated for 5 minutes with tyramide-Cy5 signal amplification reagent (Perkin-Elmer, Waltham; MA, USA). Enzyme activity was quenched by removing the slides from the Autostainer and treatment with 0.1% sodium azide for 10 minutes. For cleaved-Caspase-3, a separate set of TMA slides were incubated in 1:1500 dilution of a rabbit polyclonal anti-human antibody (Cell Signaling Technology) in Signal Stain® Antibody Diluent (Cell Signaling Technology), for 60 minutes at room temperature (RT). After 3 washes in 1x TBST, slides were incubated with the rabbit Envision+ HRP system (secondary antibody, Dako) for 60 minutes at RT. The epithelial (tumor) compartment was defined by staining with a rabbit polyclonal anti-pan-cytokeratin (PCK) antibody (Acris, San Diego, CA, USA) and an Alexa555-conjugated rabbit secondary antibody (Invitrogen, Burlington, ON, Canada). Slides were mounted using Prolong Gold Anti-fade with 4',6-diamidino-2-phenylindole (DAPI; Invitrogen). HEK293 cells overexpressing ING1 and tonsil tissue with or without primary antibody were used as controls for ING1 staining. Slides containing Jurkat cells treated with etoposide (Cell Signaling Technology) and tonsil tissue, with or without primary antibody, were used as controls for cleaved-Caspase-3 staining.

### Automated image acquisition and analysis

Automated image acquisition was performed using either the HistoRx PM-2000™ scanner (Genoptix; Carlsbad, CA, USA) for ING1 or the Aperio Scanscope® FL slide scanner (Aperio, Vista, CA, USA) for Caspase 3. Digital images were analysed using the HistoRx AQUAnalysis® software version 2.3.4.122 (Genoptix, Carlsbad, CA, USA). High-resolution monochromatic 10-bit digital images were obtained using specific filters to define the tumor (Alexa555) and nuclear (DAPI) compartments. ING1 protein levels were determined within the tumor compartment (PCK mask), tumor nuclei (DAPI within the PCK mask) and tumor cytoplasm (Tumor Mask minus DAPI). Cleaved-Caspase-3 expression in the tumor was defined as a percent-positive tumor area (% cleaved-Caspase3) calculated from the area covered by cleaved-Caspase-3 within the tumor compartment (area positive for both pan-cytokeratin and cleaved-Caspase3) and the area covered by the tumor compartment within each TMA core. % cleaved-Caspase-3 was calculated by dividing the cleaved-Caspase-3-positive tumor area by the total tumor area for each TMA core. BAX, BCL-2 and BCL-XL protein levels were determined as previously described (26). For each biomarker, the mean score from the three TMA cores was used for analyses.

### Cell lines and culture

HEK293, CAL-27 (American Type Culture Collection; ATCC, Manassas, VA, USA), UMSCC1 and UMSCC14b cell lines (gifts from Dr. Thomas Carey, University of Michigan) were used in this study. All cell lines were grown at 37°C with 5% CO_2_ in high-glucose Dulbecco's Modified Eagle Medium supplemented with 10% fetal bovine serum (Life Technologies, Burlington, ON, Canada), 0.1mg/ml streptomycin and 100U/ml penicillin (Sigma-Aldrich, Oakville, ON, Canada). Culture media were changed every 2–3 days. All cells tested negative for mycoplasma.

### Ionizing radiation treatment

Cells were plated in 6cm dishes and treated with 2, 4, 6, 8 and 10Gy doses of IR emitted by a Cesium-137 radiation source in a Gammacell® 1000 Elite irradiator (MDS Nordion, Ottawa, ON, Canada).

### Indirect immunofluorescence

Staining protocols have been described previously [1]. Microscopy was performed on a LSM 510 META confocal microscope (Zeiss; Toronto, ON, Canada).

### Adenoviral constructs

The adenoviral constructs used in this study have been previously described [29]. Viral titres were optimised to identify multiplicities of infection (MOIs) that achieved infection of >95% cells as monitored by eGFP expression. No toxicity was observed when control adenoviruses were used at these MOIs.

### Combination index calculation

Cell lines were infected with varying multiplicities of infection (MOIs) of Ad-ING1 alone or in combination with varying doses of IR. CAL27 and UMSCC1 cells were treated with IR 12hrs after infection with Ad-ING1 and harvested after 12hrs. UMSCC14B cells were treated with IR 24hrs after infection with Ad-ING1 and harvested after 24hrs. Cell viability was assessed using the 3-(4,5-dimethythiazol-. 2-yl)-2,5-diphenyl tetrazolium bromide (MTT) assay and combination indices were calculated using the CalcuSyn software (Biosoft, Cambridge, UK) as described earlier [29]. The output is represented as combination indices (CI) and isobolograms. CI values below 1.00 indicate synergy and those above 1.00 indicate antagonism.

### Western blotting

We assessed cleavage of PARP1 and Caspase-3 to confirm that cell death was mediated by apoptosis. OSCC cells infected with ING1 adenoviral constructs or adenoviral controls were washed with PBS, lysed in Laemmli's sample buffer (Bio-Rad, Mississauga, ON, Canada), sonicated on ice and aliquots containing 50μg of protein were loaded on 12.5% polyacrylamide gels and electrophoresis was performed at a constant 100V. Samples were transferred to nitrocellulose membrane (Whatman, Piscataway, NJ, USA) and probed with rabbit monoclonal anti-cleaved-caspase-3 (Cell Signaling Technology), mouse monoclonal anti-PARP1 and anti-actin antibodies (Santa Cruz Biotechnology, Santa Cruz, CA, USA).

### Statistical analysis

All experiments in this study were performed at least three times except those involving human tissue samples. X-Tile version 3.6.1 software was used to determine optimal cut-points to dichotomize continuous ING1 AQUA® scores [30]. Miller-Siegmund tests were used to formally adjust significance testing for multiple comparisons when using X-Tile software. In Table 1, Fisher's exact test was used to compare clinical covariates between the two patient groups defined by low or high cytoplasmic ING1 localization. Kaplan-Meier curves and Cox proportional hazards models were used to assess association with 5-year DSS defined earlier in result section. All statistical analyses were performed using Stata 12 (StataCorp LP, College Station, Tx, USA).

## SUPPLEMENTARY FIGURES


