# Supplementary material for: Tumor cell apoptosis mediated by cytoplasmic ING1 is associated with improved survival in oral squamous cell carcinoma patients
Source: Oncotarget. 2014 Apr 17;5(10):3210–9. doi: 10.18632/oncotarget.1907 (PMC4102804; doi:10.18632/oncotarget.1907)
Supplement: Supplementary file 1 [file oncotarget-05-3210-s001.pdf]

Tumor cell apoptosis mediated by cytoplasmic ING1 is associated with improved survival in oral squamous cell carcinoma patients.

Supplementary Information

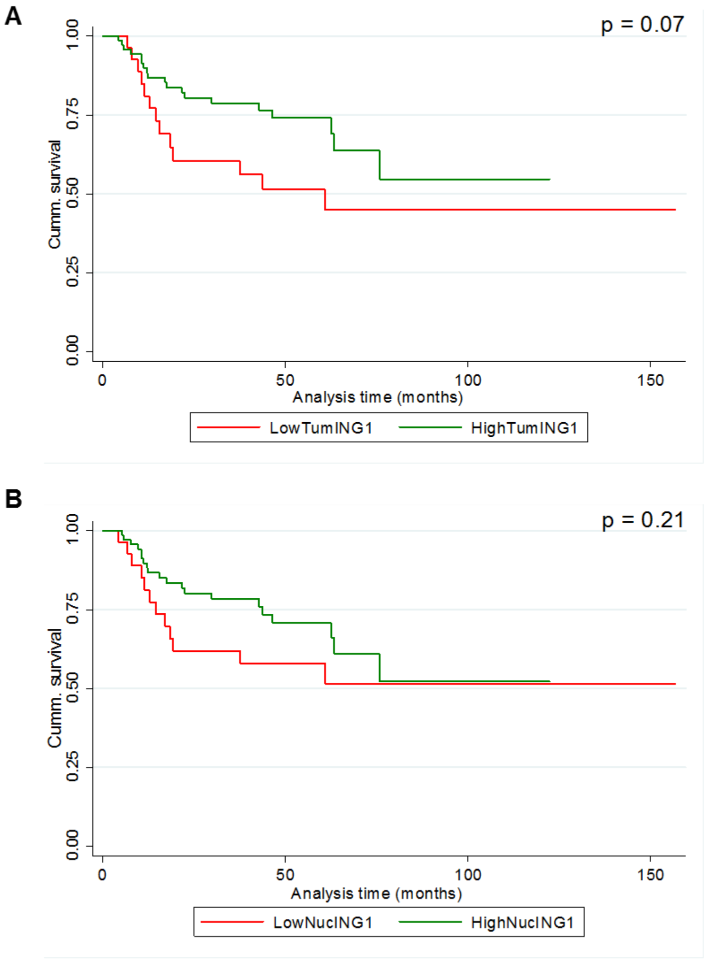

Supp Figure 1: Survival association of tumor and nuclear ING1 levels. (A) Kaplan-Meier survival curves showing association between total tumor ING1 levels and DSS. (B) Kaplan-Meier survival curves showing association between nuclear ING1 levels and DSS.

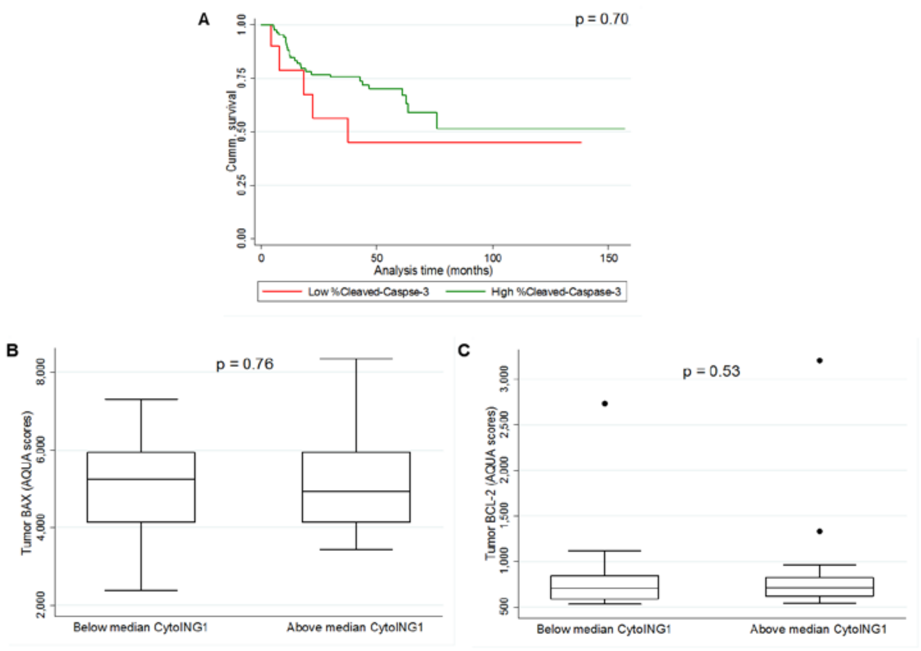

Supp Figure 2: Cytoplasmic ING1 and apoptosis. (A) Kaplan-Meier survival curves showing association between cleaved-Caspase-3 and DSS. (B) BAX protein levels compared in patients with below and above median cytoplasmic ING1 levels. (C) BCL-2 protein levels compared in patients with below and above median cytoplasmic ING1 levels.

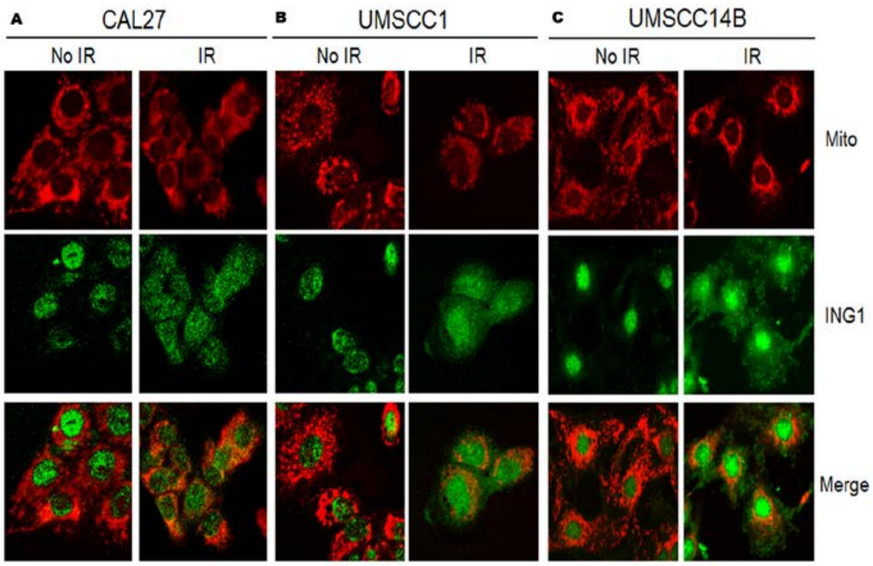

Supp Figure 3: Analysis of ING1 localization in OSCC cell lines in response to IR treatment. Representative fluorescence micrographs of untreated or IR-treated (A) CAL-27 (B) UMSCC1 and (C) UMSCC14B cells. Cells were treated with 2Gy of IR and harvested after 24hrs. Images were acquired at 63X magnification.
